# Paired Testing of Sexually Transmitted Infections With Urine Pregnancy Tests in Incarcerated Women

**DOI:** 10.1097/OLQ.0000000000001456

**Published:** 2021-05-27

**Authors:** Christine M. Dang, Julie Pao, Dena Taherzadeh, Ank E. Nijhawan

**Affiliations:** From the ∗ UT Southwestern Medical Center; † Parkland Health and Hospital Systems, Correctional Health; ‡ Division of Infectious Diseases and Geographic Medicine, UT Southwestern Medical Center, Dallas, TX

## Abstract

In correctional facilities, sexually transmitted infections (STIs) are common, are often asymptomatic, and may lead to adverse outcomes such as infertility and increased HIV acquisition. In January 2020, testing for *Neisseria gonorrhoeae* (GC) and *Chlamydia trachomatis* (CT) was paired with routine urine pregnancy tests among women entering a large county jail to increase testing and detection of STIs.

**Methods:**

The results of all GC/CT urine tests in the Dallas County Jail were collected from October 2019 to February 2020. Medical records were reviewed to collect demographic factors and to determine the positivity of GC/CT infections, time to results, and time to treatment.

**Results:**

With paired testing, monthly testing rates increased 4.7-fold among incarcerated females from 125 to 589 tests without substantial change for males (174 to 163). The number of infections detected in females increased from 25 (7%) of 359 to 62 (5.3%) of 1171 for GC and 42 (11.2%) of 374 to 129 (11%) of 1177 for CT without a significant difference in GC (*P* = 0.23) or CT positivity (*P* = 0.66). Younger women (≤25 years) had the highest rates of CT (18.8% [66/350]), whereas GC was highest among women aged 31 to 35 years (9.9% [32/321]). Average time to results and treatment were 5.8 and 1.8 days, respectively.

**Conclusions:**

Pairing GC/CT testing with routine urine pregnancy tests resulted in a large (4.7-fold) increase in the number of STI tests and a comparable increase (3.7- to 4.6-fold) in the number of infections detected. Future efforts should focus on incorporating STI testing into routine jail practices and expediting treatment to further improve outcomes in this vulnerable population.

## Abstract 2

Pairing gonorrhea/chlamydia with urine pregnancy tests in a large urban jail led to a nearly 5-fold increase in completed tests and a corresponding increase in positive test results.

Sexually transmitted infections (STIs) have become a growing epidemic in the United States in recent years. In 2018, a total of 1,758,668 chlamydial infections were reported to the Centers for Disease Control and Prevention, making it the most common notifiable STI in the United States, followed by gonorrhea with 583,405 cases. These numbers have steadily grown since 2014, with the number of cases of chlamydia rising by 19.4% and cases of gonorrhea rising by 63%.^1^ Sexually transmitted infections disproportionately affect incarcerated people, with federal prisoners being more than twice as likely to have an STI than the general population.^2^ Furthermore, a majority of incarcerated people are racial and ethnic minorities coming from disadvantaged socioeconomic backgrounds with generally poor access to health care.^3^ The ramifications of untreated STIs can be severe because these patients are at increased risk of acquiring human immunodeficiency virus (HIV), developing pelvic inflammatory disease, and potential infertility. Correctional settings have become an important point of access to health care in this population and, from a public health standpoint, can be vital not only in the detection and treatment of STIs in a high-risk population but also in the prevention of transmission to other community members.

Several previous studies have shown that when universal screening is implemented in the jail system, high rates of STIs are detected both in males and females that would have otherwise been missed.^4,5^ The Centers for Disease Control and Prevention has also emphasized the importance of STI screening in correctional settings and created guidelines specific to incarcerated individuals, advising universal screening for *Neisseria gonorrhoeae* and *Chlamydia trachomatis* (GC/CT) in all females 35 years or younger and males younger than 30 years.^6^ However, despite this recommendation, only 4% of jail systems provide mandatory or routine STI screening.^7^ New jail-based initiatives and approaches are needed to help bridge this gap between what is supported in the literature and current practice. One approach is pairing STI testing with routine laboratory work, such as a urine pregnancy test, to expand screening in the incarcerated setting. In the case of HIV, previous studies have shown that people are more likely to accept HIV testing if it is paired with routine testing on an opt-out basis.^8^ This strategy could also be applied to GC/CT testing and could reduce the negative stigma by associating it with a routine urine test required for all patients.

In this study, we aimed to implement and evaluate opt-out GC/CT testing paired with routine urine pregnancy tests for females entering the jail who are 50 years or younger. We sought to assess if our intervention would (*a*) result in completion of more STI tests than in the preceding year, (*b*) identify more positive GC/CT test results (higher absolute number) than previous testing approach, and (*c*) identify risk factors to inform more efficient screening methods in the future. We anticipate that our findings will help direct future STI testing for females in US correctional systems.

## METHODS

### Participants and Procedures

This is a prospective quality improvement study of paired GC/CT testing with routine urine pregnancy tests in women entering the Dallas County Jail (DCJ). Beginning in January 2020, women 50 years or younger who entered the DCJ received opt-out GC/CT testing performed on urine specimens that were collected for pregnancy tests. In addition, baseline retrospective data on GC/CT test results for women during the 3 months before the intervention (October–December 2019) were collected, and GC/CT testing for men was performed from October 2019 to February 2020. Data on GC/CT for men served as a comparison group to account for temporal trends in testing.

The DCJ is the eighth largest jail in the United States with an average daily census of approximately 6300 and an average of 255 admissions per day. Approximately 46% of all incarcerated people are released within 48 hours, and the average length of stay is 25 days.^9^ Beginning January 1, 2020, a standing order was automatically generated for urine GC/CT testing for all women 50 years or younger who were entering the jail. After women entering the jail undergo routine central intake procedures including triage based on medical or psychiatric issues, a nurse collects urine for point-of-care pregnancy testing. This urine sample is then sent out to an outside laboratory (LabCorp, Burlington, NC) for GC/CT testing (by nucleic acid amplification test) for females 50 years or younger. Incarcerated women 51 years and older and males are able to receive GC/CT testing upon request by the patient or provider. Test results are sent back to the jail within 3 to 5 days, scanned into the electronic health record, and forwarded to the ordering provider, who is responsible for test notification and ordering treatment as indicated.

### Data Collection

Variables collected from the DCJ electronic medical record (Pearl), include age, gender, race, GC/CT test results, and date of GC/CT results. Age was calculated at the time of the receipt of GC/CT test results. Gender was defined as sex assigned at birth, male or female. Race was coded as Black/African American, White, or other (including Asian and Native American). Individual electronic medical record review was conducted for individuals with positive test results and included whether or not treatment was ordered during incarceration, date of treatment, and length of stay.

### Primary Outcome Variables

Primary outcome was a positive test result for GC and/or CT. In addition, for patients with positive GC and/or CT test results, time to results (defined as number of days between start of incarceration and receipt of positive result), time to treatment (defined as number of days between receipt of positive result and antibiotic prescription), and length of stay were collected. Individuals who were treated during a different incarceration than when they were screened for GC/CT were not accounted for when calculating for length of stay, time to results, and time to treatment. Furthermore, individuals who were incarcerated before January 1, 2020, were not included in the calculation for time to results, as this was the date when paired opt-out screening fully went into effect.

### Statistical Analysis

Demographic characteristics (race, gender) were summarized by frequency. Age was tabulated as a range, in subsets of 5 years, with the exception of ≤25 and 51+ years old. We chose to include all individuals 51 years and older as a separate category, as this was the cutoff age used for paired urine pregnancy with urine GC/CT testing. Our main unit of analysis was GC/CT tests rather than individuals.

## RESULTS

### Testing Overall

**Figure 1:**
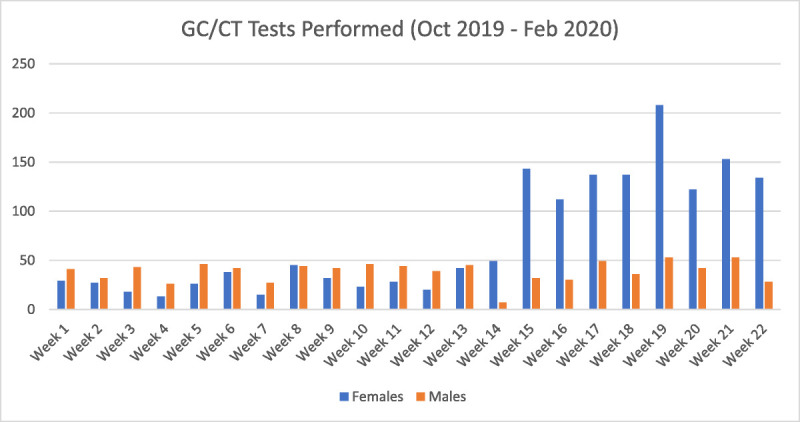
Number of GC/CT tests performed per calendar week in both incarcerated men and women from October 2019 to February 2020 (week 15 marks the beginning of opt-out testing).

During the 3 months before the initiation of opt-out testing, 374 tests were performed among females, averaging to 125 tests per month, and 522 tests were performed among males, averaging to 174 tests per month. After opt-out paired testing was implemented, 1177 tests were performed among females in January 2020 to February 2020, averaging to 589 tests per month, a 4.7-fold increase in monthly testing rates compared with the preintervention period (Fig. 1). On the other hand, testing rates in males remained stable at 326 tests in total or 163 tests per month in the postintervention period. Monthly female jail census did not vary substantially between the preintervention and postintervention periods, remaining stable at 952 and 1077, respectively. The implementation of opt-out testing corresponded to an increase in the monthly percentage of females tested from 12.7% to 54.4%; however, in males where opt-out testing was not implemented, this monthly percentage decreased from 4.3% to 3.5%. A small number of individuals received screening more than once because of multiple incarcerations and/or duplicate testing.

### Testing Results

**Figure 2:**
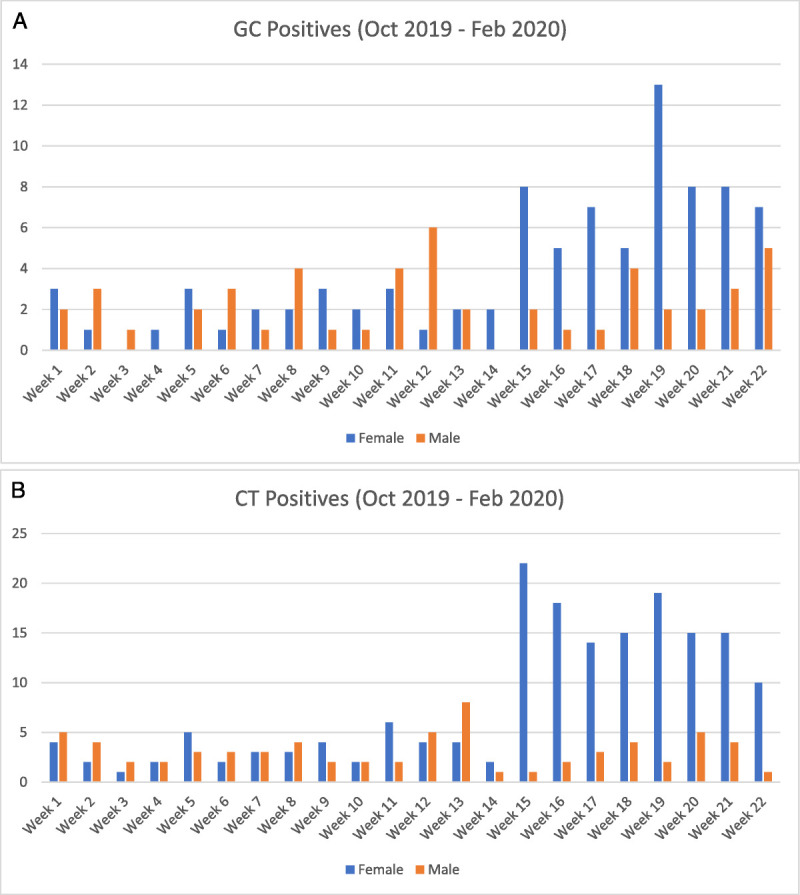
A, Number of positive GC test results per calendar week in both incarcerated men and women from October 2019 to February 2020 (week 15 marks the beginning of opt-out testing). B, Number of positive CT test results per calendar week in both incarcerated men and women from October 2019 to February 2020 (week 15 marks the beginning of opt-out testing).

Throughout the entire study period, October 2019 to February 2020, the positivity rates of GC and CT among females were 5.7% and 11.0%, respectively. For males, the GC and CT positivity rates were 5.9% and 8.0%, respectively (Table 1). After the implementation of paired testing, detection of GC and CT infections in incarcerated women rose, yielding a 3.7-fold (8.3 to 31 GC infections detected per month) and 4.6-fold (14 to 64.5 CT infections detected per month) increase in monthly GC and CT detection rates (Figs. 2A, B). In females, the positivity of GC and CT remained stable both before and after the initiation of paired testing. There was no significant difference in positivity for GC (*P* = 0.23) or CT (*P* = 0.66) between these 2 periods of time (Table 1). In males, paired testing was not implemented, and there was also no significant difference in positivity for GC (*P* = 0.82) or CT (*P* = 0.62) throughout the entire study period, October 2019 to February 2020.

**TABLE 1:** Overall Testing Rates and Positivity of GC/CT in Incarcerated Men and Women Receiving GC/CT Urine Tests in October 2019 to February 2020 (Opt-Out Testing Began January 2020)

Sex	Testing Period	Gonorrhea		Chlamydia	
		Tested (n = 2378)	Test Positive (n = 137)	Tested (n = 2399)	Test Positive (n = 239)
		No. Tested	% Positive (n)	No. Tested	% Positive (n)
Male	10/2019–12/2019	522	5.7 (30)	522	8.6 (45)
	1/2020–2/2020	326	6.1 (20)	326	7.1 (23)
Female	10/2019–12/2019	359	7.0 (25)	374	11.2 (42)
	1/2020–2/2020	1171	5.3 (62)	1177	11.0 (129)

### Risk Factors for GC/CT

In the population of incarcerated people tested for GC/CT from October 2019 to February 2020, positive test results were found in incarcerated men and women, all races and of all age groups. Women 25 years or younger had the highest positivity for CT at 18.9%, whereas women 31 to 35 years old had the highest positivity of GC at 9.7% (Table 2). Generally, for both preintervention and postintervention time periods, screening rates and the positivity of GC/CT decreased gradually as age increased. Black/African American women had the highest positivity of CT at 11.2% (60), whereas women who identified as White had the highest positivity of GC at 7.1% (45).

**TABLE 2:** Testing Rates and Positivity of GC/CT by Age Group in Incarcerated Women Receiving GC/CT Urine Tests in October 2019 to February 2020

Age, y	Gonorrhea		Chlamydia	
	Tested (n = 1530)	Test Positive (n = 87)	Tested (n = 1551)	Test Positive (n = 171)
	No. Tested	% Positive (n)	No. Tested	% Positive (n)
≤25	348	7.5 (26)	350	18.9 (66)
26–30	347	4.6 (16)	353	12.5 (44)
31–35	321	9.7 (31)	327	10.4 (34)
36–40	229	2.6 (6)	231	6.1 (14)
41–45	142	2.8 (4)	143	3.5 (5)
46–50	103	2.9 (3)	104	4.8 (5)
51+	40	2.5 (1)	43	7.0 (3)

After paired GC/CT testing began in January 2020, the average time to results was 5.8 days (with an SD of 3.8 days). The average time to treatment was 1.8 days (SD of 1.8 days). Of the 151 females diagnosed with either GC or CT during this period, 56% (84) received treatment, whereas 44% (67) did not because of results being unavailable at the time of release. Length of stay for treated individuals was an average of 29.7 and a median of 21.5 days. The proportion of females receiving treatment before the implementation of opt-out testing was higher at 81%.

## DISCUSSION

This study demonstrates that paired screening of GC/CT with routine urine pregnancy test leads to an increased number of completed tests and a higher detection of GC and CT infections that would have otherwise been missed. Compared with testing rates before paired testing where GC/CT tests were ordered based on clinical suspicion, implementation of paired testing increased monthly testing rates by 4.7-fold. There was a similar increase in the number of positive test results after paired testing and no significant difference in positivity of GC/CT compared with before the implementation of opt-out testing (*P* < 0.001), suggesting a high rate of asymptomatic disease.

Previous studies that have also implemented opt-out screening for GC/CT have similarly reported substantial increases in GC and CT detection rates. One study in New York City implemented universal testing among males 35 years or younger housed in correctional facilities. Within the first year of testing, there was an increase of 1636% in the number of CT cases in incarcerated males, whereas detection of CT in other settings such as STD clinics and physician offices remained stable.^4^ One study from the Cook County Jail implemented opt-out screening in incarcerated women and increased monthly detection rates of GC and CT by 4-fold (from 9.3 to 40.8 cases per month).^5^ Conversely, a different study in Cook County demonstrated that discontinuing universal GC/CT screening in incarcerated men led to a large decrease in the number of reported GC and CT cases reported in the jail. In the jail, GC and CT cases decreased by −90.5% and −91.7%, respectively; citywide, GC and CT cases decreased by −12.9% and −9.3%. These numbers underscore the substantial and far-reaching impact of STI screening in correctional facilities.^10^

Other studies that have implemented opt-out screening of GC/CT in incarcerated females have similarly detected high GC/CT prevalence compared with the general population. The positivity of GC found in our study (5.3%) was higher than rates previously identified in other incarcerated female populations (2.5%–3.1%), whereas the positivity of CT found in our study (11.0%) was consistent with previously reported rates (7.6%–11.4%).^5,11^ Overall, GC/CT prevalence detected in the incarcerated population is substantially higher compared with that of the general US population, estimated to be at 0.34% and 2.0%, respectively, among females 14 to 39 years old.^12,13^

Our findings demonstrate an inverse relationship of GC/CT infection with age in females, which is consistent with the prevalence found in other incarcerated populations and the community.^5,14,15^ In our study, younger females (≤25 years old) had the highest positivity of CT at 18.9%, whereas women 31 to 35 years old had the highest positivity of GC at 9.7%. The main goal of this study was to evaluate the impact of paired testing of GC/CT with routine urine pregnancy tests on number of completed tests as well as the detection and treatment of GC/CT during incarceration. Although evaluating the impact of jail-based screening on community GC/CT prevalence was beyond the scope of this project, previous studies have shown that increasing STI testing in correctional settings can have a beneficial impact in the community. In 2009, jails in the San Francisco metropolitan area implemented opt-out testing for incarcerated men, which corresponded with a decrease in female GC/CT positivity in community clinics located in neighborhoods heavily impacted by incarceration (and no impact in STD clinics in other local areas), suggesting a decrease in community transmission after incarceration.^16^ Individuals who are or who have previously been incarcerated are estimated to represent 24% of all STIs in the US population and generally have limited access to health care.^17^ Consequently, the correctional setting represents an important opportunity to screen incarcerated people for STIs, and to identify and treat these infections, in both men and women. Because counties with higher incarceration rates have also been associated with higher rates of GC and CT, the expansion of screening efforts through paired testing could represent the first step in decreasing these geographic disparities.^18^

More widespread GC/CT screening in incarcerated populations has been repeatedly shown to increase detection of otherwise unknown GC/CT infections.^4,5,19^ However, despite these high detection rates, high treatment rates were difficult to obtain in this study because test results were often not available before the release of these individuals from jail. In fact, treatment rates of positive GC/CT infections decreased from 81% to 56% upon the initiation of opt-out testing. This was likely because women were being testing earlier during incarceration, leading to a higher number being released before results are available. Informing individuals of their test results and linking them to treatment were also difficult because addresses recorded in the jail system were not always accurate. Expediting results could potentially increase treatment rates during incarceration, and improving transitions of care outside of the jail could help released individuals become connected with treatment after their release. For individuals who did receive treatment, they remain at risk for reinfection when they reenter the community and engage with previous sexual partners. To further limit the community spread of STIs, some programs have adopted patient-delivered partner therapy, which entails providing patients diagnosed with a GC/CT infection medication to take to his/her partner, although this has not been evaluated among individuals released from correctional facilities and merits further study.^20^

This study has a number of limitations. Information about symptoms was not available as part of this project, minimizing our ability to determine specifically what proportion of GC/CT infections were asymptomatic and would otherwise have been missed. Furthermore, ethnicity (e.g., Latinx) was not available for this project, limiting the opportunity to identify or interpret differences in the detection or treatment of GC/CT by racial/ethnic group. For instance, White females in this study had the highest positivity of GC at 7.1%; however, according to national data, African American/Black females have had much higher rates compared with other groups.^21^ This discrepancy could be due to the lack of delineation between White and Hispanic females in our data. We were also unable to assess the successful notification and treatment of GC/CT infections once patients were released into the community. However, certified letters were mailed to patients informing them of an abnormal laboratory result and the Dallas County Health and Human Services Department was notified of any patient with a positive GC or CT result who was released before receiving treatment. In addition, because gender in DCJ records is based on sex assigned at birth rather than self-reported gender, our results could have limited applicability to trans and nonbinary persons. Future directions of this project would be to identify opportunities to expedite STI diagnoses and delivery of treatment during incarceration, expand STI testing to incarcerated men who represent approximately two-thirds of the DCJ population and a large pool of undetected disease, and improve care coordination for patients after jail release. Although duration of incarceration in jail is often relatively short, increasing sexual health education soon after jail entry may improve follow-up with STI treatment and engagement in primary care services such as HIV preexposure prophylaxis.

Correctional facilities provide an important venue for implementing medical interventions, as providers can care for a patient population that may have limited access to health care. In our study, pairing screening of GC/CT with routine urine pregnancy tests not only increased the number of tests performed but also increased the number of GC/CT infections detected by nearly 5-fold. Continuing and optimizing paired testing among incarcerated women and expanding screening among incarcerated men has the potential to have a major impact on the health of this vulnerable population and the general community.
